# Epidemiological evaluation of cholera outbreak dynamics in conflict-affected Central and Northwestern Tigray, northern Ethiopia: evidence from GEE analysis

**DOI:** 10.1017/S0950268826101514

**Published:** 2026-04-30

**Authors:** Haftu Gebrehiwot Gebremeskel, Seyoum Desta, Senait Haddis Abebe, Mebrahtom Hafte Amaha, Equbay Gebregziabher Gebru, Goyitom Gebremedhn, Michele Hagos Debesay, Rieye Esayas Belay, Kibrom Gebrehiwot Gebremeskel

**Affiliations:** 1 Biostatistics, Mekelle University, College of Natural and Computational Sciences, Mekelle, Tigray, Ethiopia; 2 Department of Biostatistics, Public Health Researcher and Data Reviewer, Ethiopia; 3 Reproductive Health, Tigray Health Research Institute, Mekelle, Tigray, Ethiopia; 4 Reproductive Health, Tigray Regional Health Bureau, Mekelle, Tigray, Ethiopia; 5 Department of Mathematics, Aksum University, Axum, Ethiopia

**Keywords:** case fatality rate, cholera, epidemiological analysis, Generalized Estimating Equations, outbreak

## Abstract

Cholera remains a major public health concern in conflict-affected Tigray, Ethiopia, where disrupted water, sanitation, and hygiene (WaSH) services and displacement have increased transmission risk. This study analysed outbreak dynamics, attack rates (AR), and predictors of cholera to inform interventions aligned with Ethiopia’s Cholera Elimination Plan (2022–2028). A retrospective analysis was conducted on 802 suspected and confirmed cholera cases reported from 25 July to 4 October 2024 across 25 districts in Central and Northwestern Tigray. Data from the Tigray Health Research Institute line list were analysed using descriptive statistics, Chi-square tests, and Generalized Estimating Equation (GEE) models. Attack rates were highest in Asgede (357.25/100000) and Endabaguna (88.12/100000). Significant associations were observed with age, sex, occupation, water source, travel history, vaccination, latrine access, and contact history. GEE analysis showed strong intra-cluster correlation (α = 0.949). Higher odds of cholera were associated with males, adults aged 16–45 years, and use of unsafe water sources, while vaccination and latrine availability reduced risk. Strengthening WaSH services, vaccination coverage, surveillance, and targeted risk communication is essential to reduce cholera transmission in Tigray.

## Introduction

Cholera is an acute intestinal infectious disease caused by toxigenic strains of the bacterium *Vibrio cholerae* (*V. cholerae*) of the *O1* and/or *O139* serogroups [1–3]. Transmission occurs primarily through ingestion of contaminated water or food via the faecal–oral route. *V. cholerae* can also persist naturally in aquatic environments such as brackish rivers, estuaries, and coastal waters, often in association with plankton and shellfish, allowing infection to occur through contaminated environmental water or aquatic food sources [4–6]. The disease is characterized by the rapid onset of profuse watery diarrhoea that can lead to severe dehydration and death if untreated [7].

The case fatality rate (CFR) for untreated cholera can exceed 50%, but prompt treatment with rapid oral or intravenous rehydration can reduce mortality to below 1%. Antibiotics are recommended only for patients with severe dehydration or severe disease, as they shorten the duration of diarrhoea and reduce bacterial shedding [1]. However, in low- and middle-income countries and in settings affected by natural disasters or humanitarian crises, access to timely and appropriate treatment, including antibiotics when indicated, is often constrained by fragile health systems and inadequate water, sanitation, and hygiene (WaSH) infrastructure [2, 8–10].

Globally, cholera remains a significant public health challenge [11], with 733956 cases and 5162 deaths reported between 1 January and 24 November 2024 [12]. The Eastern Mediterranean Region accounted for the majority of cases (554434; eight countries), followed by the African Region (150156; 18 countries) [11, 12]. In November 2024 alone, 58749 new cases were reported globally, a slight decrease from October, though still 37% higher than November 2023 [11, 12]. Sub-Saharan Africa has experienced a resurgence, with 14 countries affected in 2024, reporting 112301 cases and 1900 deaths (CFR 1.7%) between January and July. Five countries, Comoros, DRC, Ethiopia, Zambia, and Zimbabwe, contributed over 85% of the cases and deaths [11, 12]. According to the Ethiopian Public Health research Institute (EPHI) national cholera line-list, Ethiopia has been subjected to recurrent cholera outbreaks over the past 9 years (2015–2023), reporting nearly 100000 cases and over 1000 deaths between 2015 and 2023 [13, 14]. A significant outbreak in 2022 resulted in 841 cases (CFR 3.13%), escalating to over 30000 cases in 2023 with a CFR of 1.4% [14]. By May 2024, more than 46800 cholera cases had been reported across 78 districts in eight regions of Ethiopia [15]. The country’s National Cholera Elimination Plan (2022–2028) aims to achieve zero local transmission in hotspot areas by 2028 [12, 16]. Tigray, identified as one of Ethiopia’s regions prone to seasonal cholera outbreaks [16], has faced significant challenges due to conflict, widespread displacement, ravaged health and WaSH facilities, disrupted health services, limited healthcare access, and overcrowded conditions in internal displacement and refugee camps [17–19].

Waterborne diseases like cholera flourish in these conditions due to limited and disrupted access to clean water, poor sanitation, and overstretched healthcare services. The disruption to water supply systems, particularly in urban areas, coupled with the absence of basic sanitation facilities in IDP camps, has heightened the risk of cholera outbreaks [20–23]. In July 2024, there was an alarming re-emergence of a cholera outbreak in several accessible zones of the Tigray region, including Mekelle, Central, Eastern, and Northwestern.

The outbreak is exacerbated by several environmental and social factors [24]. Data collected from 31 July to 26 August 2024 show that 86% of cholera patients use unsafe river water for drinking purposes and only 6% report the use of latrines. Severely dilapidated IDP sites in and around Shire were identified at high risk of cholera several months ago, as WaSH services were not provided for more than 6 months, leaving people without access to safe drinking water and appropriate sanitation [25]. High-risk groups, including daily labourers in remote gold mining areas with no access to safe drinking water or proper hygiene, have further fuelled the spread. Widespread misconceptions about cholera, poor awareness, and confusion at health checkpoints have hindered control efforts.

**Figure 1.**
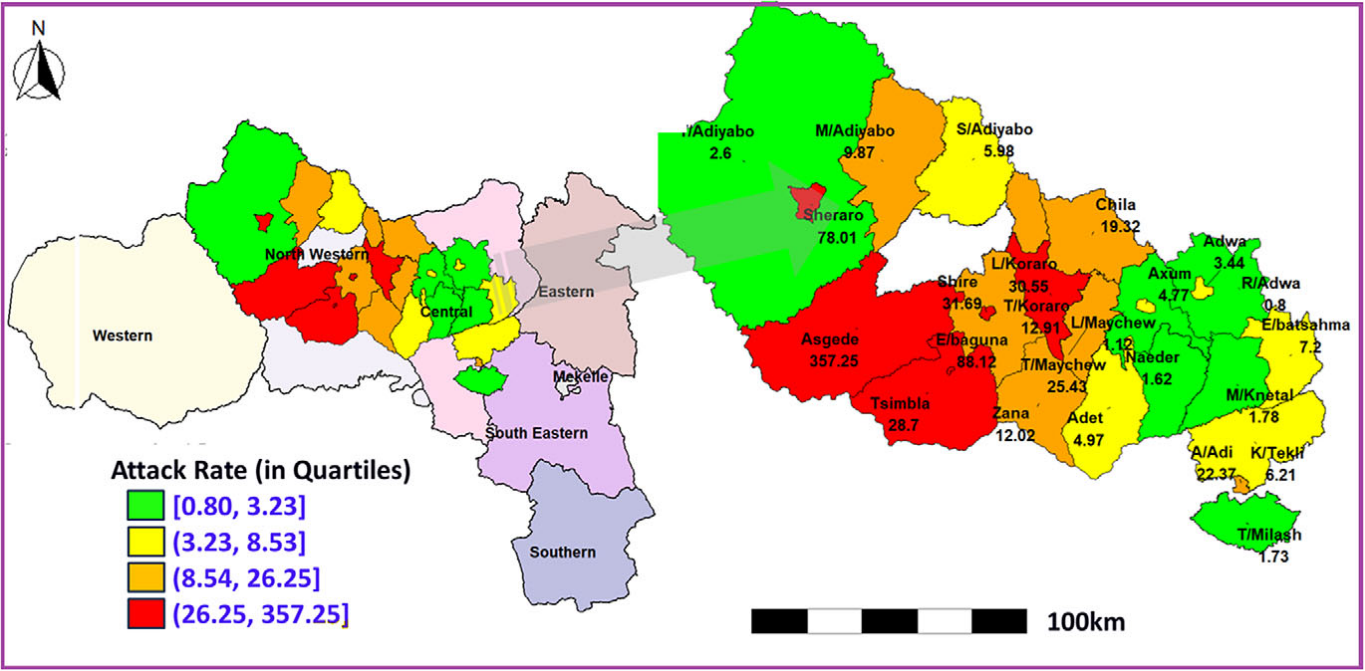
Quartile-based classification of cholera attack rates across districts in Central and Northwestern Tigray.

Inadequate Cholera Treatment centres and Units, coupled with floods (in Shire) causing latrine overflows, have exacerbated the crisis, leaving many vulnerable communities at high risk. The cholera outbreak in Tigray entails a great attention, as it poses an additional layer of threat to a population already weakened and devastated by conflict, malnutrition, displacement, and economic downturn. As part of the effort to change this equation and achieving the National Cholera Elimination Plan (2022–2028), this comprehensive epidemiological analysis of the cholera outbreak in the Tigray region was conducted based on 802 cholera cases (suspected and confirmed), amassed from 25 July 2024 to 4 October 2024 to characterize the outbreak, estimate attack rates, and identify risk factors across 25 districts in the two most affected zones (Central and Northwestern) among the six accessible zones of Tigray, with one zone (Western zone) remaining inaccessible (Figure 1).

The evidence-based conclusion and recommendations synthesized from the study could be used to strengthen public health preparedness, interventions, and response strategies for future cholera outbreaks in the region.

## Methods

### Study setting

Tigray, the northernmost regional state of Ethiopia, has been heavily affected by armed conflict since November 2020. The region is administratively divided into seven zones: Mekelle (regional capital), Southern, Southeastern, Central, Eastern, Northwestern, and Western. These zones are further subdivided into 93 districts and 814 kebeles (*lower government’s administrative structure below the District*) [26]. Tigray is bordered by Eritrea to the north, Amhara to the south, Afar to the east, and Sudan to the west [27]. The region covers an area of 53386.18 km^2^ and had a projected population of 7.3 million in 2022, although this may have changed due to the conflict [28]. During the study period, six of the seven zones were accessible for surveillance and response activities, while Western Tigray remained inaccessible due to security constraints. This study, therefore, focused on the two most affected accessible zones, Central and Northwestern Tigray.

### Study design and population

We conducted a retrospective epidemiological study using cholera outbreak data reported between 25 July and 4 October 2024 in the most affected accessible zones of Tigray. The study population included all cholera cases reported in the Central and Northwestern Zones of Tigray, Ethiopia, during this period. This encompassed both suspected and confirmed cholera cases, including inpatients, outpatients, individuals treated within the community, and those identified through active surveillance. The study also accounted for patients who recovered as well as those who died before or during hospitalization.

### Data source

We obtained secondary data from the cholera line list (CLL) maintained by the *Tigray Health Research Institute* (THRI). The line list is compiled through the routine public health surveillance system during outbreak response. Health facilities report suspected and confirmed cholera cases using standardized reporting forms, which are aggregated from health posts and health centres to district and zonal levels before being consolidated at the regional level. Surveillance teams verify reported cases through facility records, laboratory reports, and field investigation during active case finding. Reporting was conducted on a daily basis during the outbreak period. Case-based surveillance included both passive reporting from health facilities and active case finding conducted by outbreak investigation teams. Patients were followed until discharge or death, and outcomes were updated in the surveillance database. Geographic location reflects the patient’s place of residence rather than the reporting health facility. Risk factor information was collected using the WHO CLL investigation form by the Task Force working on the Cholera Control Surveillance Working Group. Data were collected through structured interviews with patients or caregivers at treatment facilities and during household investigations. Variables included demographic characteristics, water source, sanitation access, household disinfection, travel history, and contact with suspected cases. Population denominators used to calculate attack rates and incidence proportions were obtained from the *Tigray Regional Health Bureau* (TRHB) and the *Ethiopian Public Health Institute* (EPHI) population projections based on the 2014 population estimates.

### Study variables

Cholera occurrence (illness status) was defined as a binary outcome variable for the regression analyses. All 802 participants included in the study were initially recorded in the surveillance line list as suspected or confirmed cholera-related cases. For analytical purposes, these individuals were reclassified into *cholera-positive (‘Yes’)* and *cholera-negative (‘No’)* groups based on the study case definition. Participants were classified as *cholera-positive (‘Yes’)* if they met the operational case definition for cholera, which included the presence of compatible clinical symptoms (e.g., acute watery diarrhoea [AWD]), epidemiological linkage to a confirmed case or outbreak setting, and/or laboratory confirmation (e.g., rapid diagnostic test [RDT] or culture identifying *V. cholerae*). Participants were classified as *cholera-negative (‘No’)* if they did not meet the above criteria, including individuals with negative laboratory results or insufficient clinical and epidemiological evidence to support a cholera diagnosis. Rapid diagnostic test (RDT) results were available for a subset of participants and were used as supportive evidence for case classification. Of the 802 participants, *602 (75.1%) underwent RDT testing*, while *200 (24.9%) did not*. However, cholera status was not determined solely based on RDT results; rather, it was assigned using a combination of clinical, epidemiological, and laboratory information. This binary outcome is essential to the study, allowing for a focused analysis on factors associated with cholera occurrence and providing a clear distinction between affected and unaffected individuals within the outbreak context.

The main factors associated with cholera cases (yes/positive, no/negative) are grouped into demographic, environmental/exposure, and behavioural categories. Demographic factors include age group (≤15, 16–30, 31–45, 46–60, 61+), sex (male, female), occupation (e.g., farmer, student, merchant), and geographic location (zones, districts). Environmental and exposure factors cover water sources (e.g., river, pond, pipe), sanitation facilities (latrine availability), household disinfection, contact with infected individuals, and travel history to affected areas. Behavioural and treatment factors include vaccination status, delay in seeking treatment, and hygiene practices. In the surveillance line list, occupation and displacement status were captured within a single variable. The category ‘internally displaced person’ (IDP) was retained in the analysis because displacement status reflects important contextual exposures such as crowding, mobility, disrupted living conditions, and limited access to water, sanitation, and healthcare during the outbreak. Similarly, household disinfection referred to reactive disinfection conducted by outbreak response teams after identification of a suspected or confirmed cholera case in the household, rather than a pre-existing preventive practice.

### Case definitions and surveillance procedures

We defined a suspected cholera case as any person aged 5 years or older presenting with AWD and dehydration or death from AWD. In areas where an outbreak had already been confirmed, we considered any person with AWD, regardless of age, as a suspected case. We defined a confirmed case as a suspected case with laboratory confirmation of *V. cholerae O1* or *O139* through culture or rapid diagnostic testing performed in designated laboratories. Laboratory confirmation was conducted using available regional laboratory capacity, supported by national reference laboratory procedures during the outbreak response.

Active surveillance was implemented through health facilities and community health workers who conducted case finding in affected districts, including visits to communities, displacement sites, and high-risk settings. Surveillance teams routinely searched for unreported cases, verified rumours of outbreaks, and facilitated referral and reporting. These procedures were implemented to reduce under-ascertainment and improve completeness of case reporting despite the fragile health system context.

### Statistical method and analysis

Univariate descriptive and multivariable Generalized Estimating Equations (GEE) approaches were employed to analyse cholera outbreak data in these targeted zones of Tigray Region. Univariate analysis was used to summarize and describe demographic variables (such as age, gender, and occupation), clinical outcomes (such as deaths and recoveries), and environmental factors (such as water source and sanitation access) using frequencies, percentages, means, and standard deviations. Spatial analysis techniques were also applied to visualize the distribution of cholera across zones and identify high-risk areas. Associations between categorical variables (e.g., gender, zone, water source) and cholera incidence were assessed using bivariate Chi-square or Fisher’s exact tests. To model the relationship between risk factors and cholera occurrence across zones, we used Generalized Estimating Equations (GEE), which account for within-cluster correlations in clustered data [29, 30]. The cholera case data are organized by districts, with individuals within each district nested under broader zones, such as the Central zone or the Northwestern zone. Within these zones, individuals are likely to experience similar environmental exposures, share comparable social characteristics, or have access to similar infrastructural resources. These shared conditions, which may include water source quality, sanitation facilities, or healthcare accessibility, can contribute to dependencies or correlations in the cholera outcomes among individuals within the same district or zone. As a result, the outcomes are not independent but rather influenced by the common factors present within their respective geographical or administrative clusters. GEE, unlike standard logistic regression (which assumes independence of observations), accommodates this intra-cluster correlation, producing robust standard errors and reliable *p*-values. The GEE model provides population-averaged (marginal) estimates that represent the association between predictors and cholera risk across zones, using a logit link function for the binary outcome variable (cholera presence/absence). We selected an exchangeable correlation structure, which assumes uniform correlation within districts (similar environmental, behavioural, or healthcare access conditions that could influence the likelihood of cholera), reflecting our hypothesis that factors affecting cholera risk may be similar for individuals within the same zone. This approach provides population-level estimates, offering a broad view of cholera risk factors relevant for public health interventions. In contrast, logistic regression would yield subject-specific effects, which could underestimate standard errors in clustered data, leading to potentially misleading inferences. The GEE model for binary outcome, 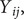 ( 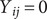 for no cholera, 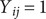 for cholera), can be expressed as follows:

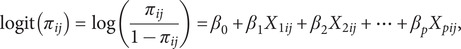

where 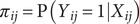 represents the probability of cholera occurrence for the *j^th^* individual in the *i^th^* zone. The terms 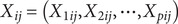 denote the predictor variables, such as sex, age group and water source. 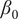 is the intercept, while 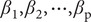 are the coefficients corresponding to each predictor, capturing the effect of each variable on the probability of cholera occurrence. Model performance was assessed using the quasi-likelihood under the independence model criterion (QIC) and robust standard errors. Discrimination was evaluated using the area under the receiver operating characteristic curve, and calibration was assessed using the Brier score. Multicollinearity among predictors was examined using variance inflation factors. To assess the impact of clustering, a standard logistic regression model was also fitted and compared with the GEE results. Sensitivity analyses were conducted to assess the robustness of the GEE model results. Alternative working correlation structures (independence and unstructured) were evaluated, and a finite-sample correction was applied to the robust standard errors to account for the relatively small number of clusters. The consistency of the estimated associations across these specifications was examined. Spatial analysis techniques, including Moran’s I and Getis-Ord Gi* hotspot analysis, were used to identify clusters of cholera incidence. Temporal trends were analysed to assess the progression of cases, deaths, and recoveries over time. Data management and analysis were conducted using SPSS (version 26) and R (version 4.3.3), with initial data entry in Excel. Results were presented in tables and figures, and statistical significance was assessed at a *p*-value <0.05.

## Results

Our analyses included 802 suspected and confirmed cholera cases reported from 25 districts in the affected accessible zones of Tigray (14 districts in the Central zone and 11 in the Northwestern zone). The findings are presented below, including spatial and temporal patterns, attack rates, and epidemiological characteristics of the outbreak.

### Disease epidemiology

*Attack rate*: The spatial distribution map in Figure 1 shows substantial variation in cholera attack rates across districts in Central and Northwestern Tigray [31], with quartile-based colour gradients illustrating the intensity of the outbreak. Districts in the highest quartile (red) represent the most severely affected areas. In Northwestern Tigray, Asgede (357.25 per 100000) and Endabaguna Town (88.12 per 100000) recorded the highest attack rates, indicating intense transmission. Other districts such as Shiraro (78.01 per 100000) and Shire Endasilassie (31.65 per 100000) also fall within higher quartiles, reflecting substantial disease burden, although lower than the peak observed in Asgede. In contrast, districts in the Central zone generally exhibited moderate to lower attack rates, mostly falling within the middle and lower quartiles (yellow and green). For example, Tahtay Maychew (25.43 per 100000) and Abi Adi (22.37 per 100000) showed moderate levels of cholera incidence. Overall, the quartile-based classification highlights clear spatial clustering of high attack rates in Northwestern Tigray, while Central Tigray experienced comparatively lower but still notable transmission.

**Figure 2.**
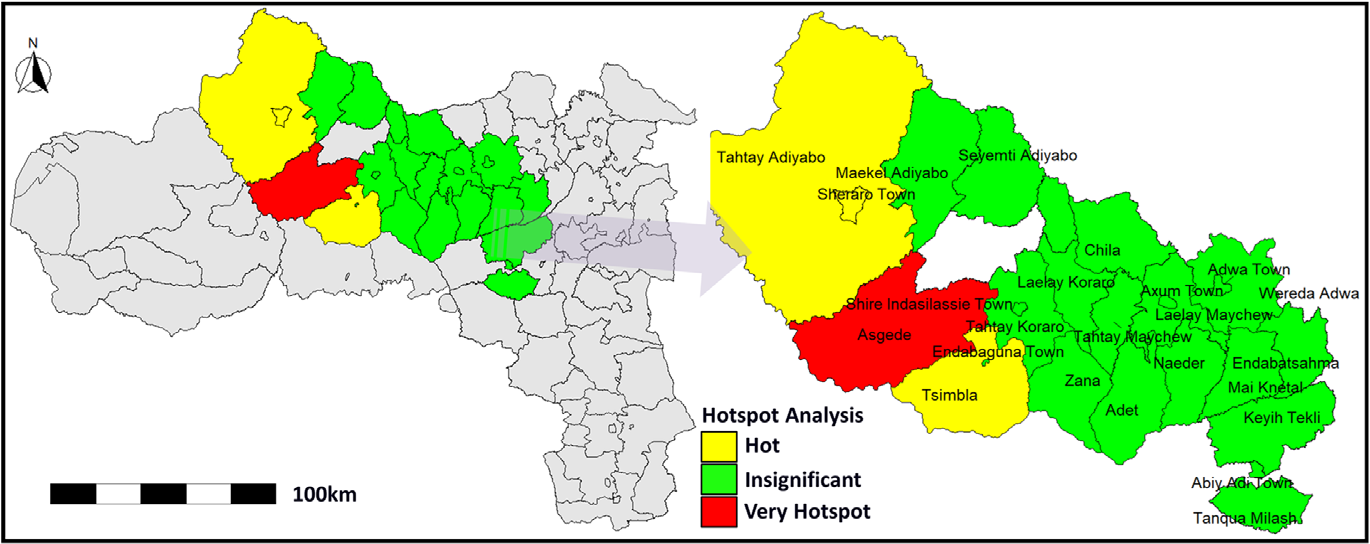
Getis-Ord Gi hotspot analysis in Central and Northwestern Tigray, Ethiopia.

*Hotspot Detection*: The Global Moran’s I value of 0.492 (*p* = 0.0018) indicated significant positive spatial autocorrelation, confirming clustering of cholera cases rather than random distribution. The Getis-Ord Gi* analysis (Figure 2) identified very high-intensity hotspots (red) and hotspots (yellow) with *p* < 0.05, while green areas showed no statistically significant clustering. The most prominent hotspots were observed in Asgede, Shire Endasilassie, and Tsimbla in the Northwestern zone, with surrounding districts showing lower transmission levels.

**Figure 3.**
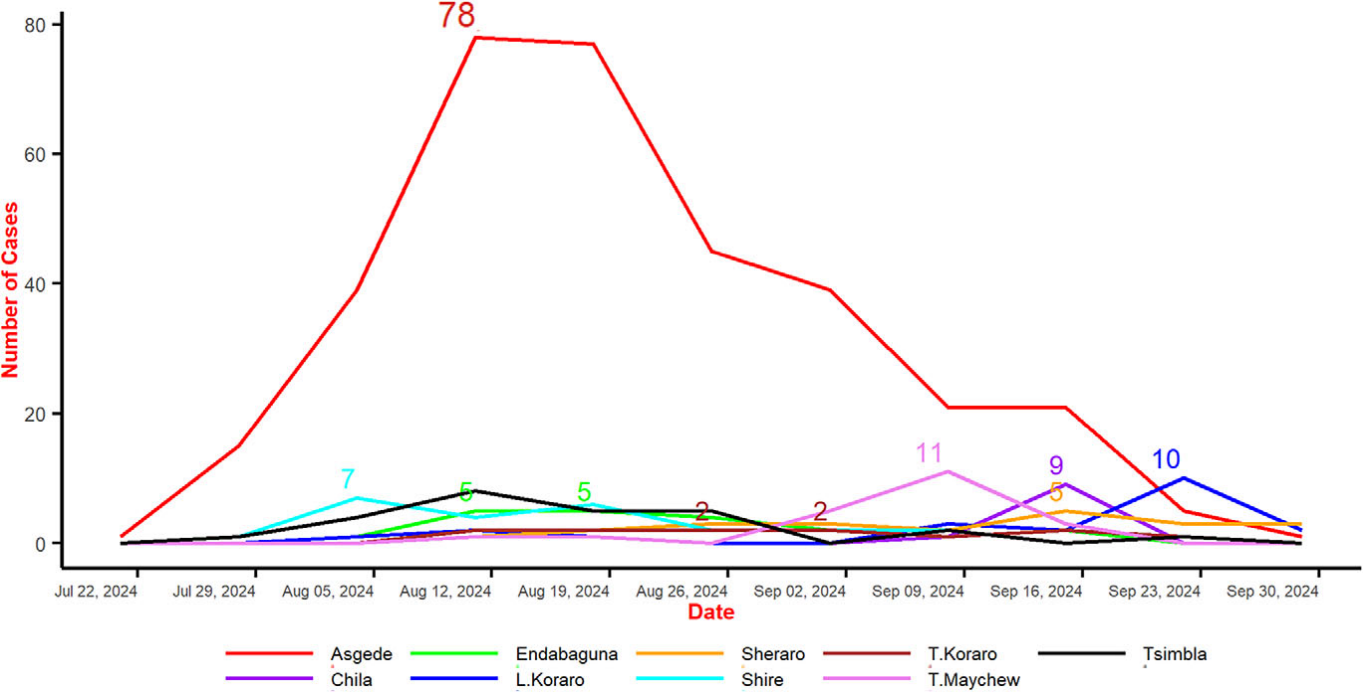
Cholera cases for most affected woredas in C-NE, Ethiopia.

*Epidemiological Trends*: The line graph in Figure 3 illustrates the progression of cholera cases across the most affected districts from late July to late September 2024. Asgede exhibited the sharpest surge, peaking at 78 confirmed cases in mid-August before declining steadily. In contrast, districts such as Endabaguna, Sheraro, Tahtay Koraro, Chila, Shire, Tahtay Maychew, and Tsimbla experienced only small, sporadic outbreaks, with case counts generally between 2 and 11. By the end of September, the epidemic curve across all districts had flattened, indicating that the cholera outbreak was largely under control.

**Figure 4.**
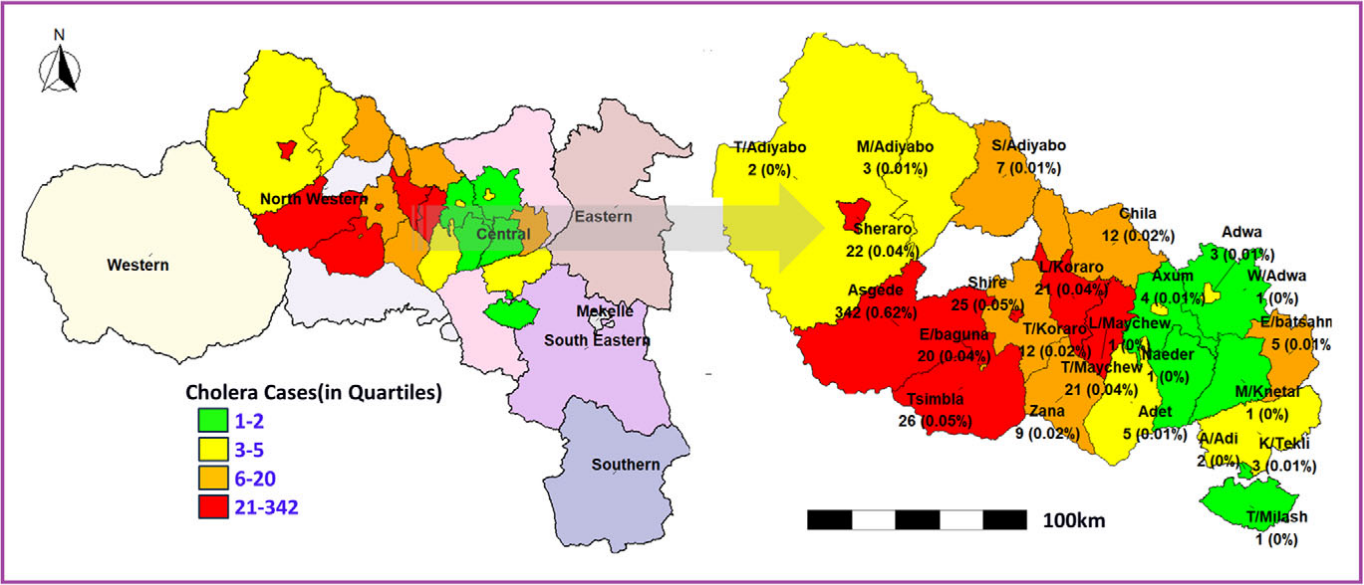
Quartile-based classification of cholera cases for most affected woredas in Central and Northwestern Tigray, Ethiopia.

*Cholera cases*: Figure 4 illustrates the spatial distribution of reported cholera cases across the study districts using a quartile-based classification. Districts in the highest quartile (red) represent the areas with the greatest burden of cases. In Northwestern Tigray, Asgede (342 cases; 0.62%) recorded the highest number of cases, followed by Tsimbla (26 cases; 0.05%), Shiraro (22 cases; 0.04%), and Shire (21 cases; 0.04%), indicating a clear concentration of cases in this zone. Moderate case counts (orange and yellow categories) were observed in districts such as Chila (12 cases; 0.02%), Zana (9 cases; 0.02%), and Tahtay Maychew (21 cases; 0.04%), suggesting ongoing but less intense transmission. In contrast, most districts in the Central zone, including Axum, Adwa, Aksum, Hawzen, and Mekelle, reported low case counts, generally falling within the lowest quartile (green), indicating limited transmission. The quartile-based classification reveals a strong spatial clustering of cholera cases in Northwestern Tigray, with a clear west-to-east gradient of decreasing burden. This pattern highlights the disproportionate impact of the outbreak in specific districts and underscores the need for targeted intervention strategies in identified hotspot areas.

### Demographic, environmental, WaSH, and health-related characteristics

Table 1 reveals the demographic, environmental, WaSH, and health-related characteristics of the study participants (*n* = 802), all of whom were enrolled from the CLL as suspected or confirmed cholera-related records. For analytical purposes, participants were classified into cholera-positive and cholera-negative groups based on the study case definition, with 554 (69.1%) classified as cholera-positive and 248 (30.9%) as cholera-negative. Significant associations were found between cholera status and several variables, including sex, age, reported zone, occupation, water source, contact with suspected AWD patients, travel history, vaccination status, latrine availability, and household disinfection. Sex was significantly associated with cholera status (*χ*^2^ = 4.30, *p* = 0.038), with females showing a higher infection rate (79.3%) compared to males (70.5%). Age distribution also demonstrated a significant relationship with cholera status (*χ*^2^ = 11.38, *p* = 0.023), the 1- to 15-year age group exhibiting the highest infection rate (82.6%).

**Table 1.** Demographic and health-related characteristics of infected and uninfected study participants (*n* = 802)

Predictors	Categories	Cholera status		χ^2^	*P*-value	Predictors	Categories	Cholera status		χ^2^	*P*-value
		No (*n* = 248)	Yes (*n* = 554)					No (*n* = 248)	Yes (*n* = 554)		
Sex (*n* = 802)	Female	28 (20.7%)	107 (79.3%)	4.30	0.038	Water source (*n* = 802)	Hand pump	26 (18.4%)	115 (81.6%)	25.74	<0.001
	Male	197 (29.5%)	470 (70.5%)				Pipe water	6 (30.0%)	14 (70.0%)		
Age group (*n* = 802)	1–15 years	19 (17.4%)	90 (82.6%)	11.38	0.023		Other	11 (33.3%)	22 (66.7%)		
	16–30 years	153 (31.9%)	326 (68.1%)				River	156 (34.2%)	300 (65.8%)		
	31–45 years	24 (23.5%)	78 (76.5%)				Spring water	1 (5.9%)	16 (94.1%)		
	46–60 years	18 (24.0%)	57 (76.0%)				Surface water	25 (18.5%)	110 (81.5%)		
	61+ years	11 (29.7%)	26 (70.3%)			Contact with suspected AWD patient (*n* = 802)	No	188 (28.0%)	484 (72.0%)	0.91	0.031
Reported zone (*n* = 802)	Central	1 (1.3%)	74 (98.7%)	34.16	<0.001		Yes	37 (28.5%)	93 (71.5%)		
	Northwestern	224 (30.8%)	503 (69.2%)			Travel history for active area (*n* = 797)	No	201 (29.4%)	484 (70.9%)	2.84	0.042
Occupation (*n* = 802)	Child	0 (0.0%)	20 (100.0%)	30.42	<0.001		Yes	24 (21.1%)	93 (81.6%)		
	Farmer	64 (23.5%)	208 (76.5%)			Time b/n symptom onset and arrival at a HF (*n* = 802)	<1 day	71 (27.7%)	185 (72.3%)	1.92	0.588
	Gold miner	117 (34.7%)	220 (65.3%)				<2 days	91 (27.2%)	244 (72.8%)		
	Herdman	3 (21.4%)	11 (78.6%)				<3 days	40 (33.1%)	81 (66.9%)		
	Housewife	0 (0.0%)	11 (100.0%)				> = 3 days	23 (25.6%)	67 (74.4%)		
	IDP	17 (37.8%)	28 (62.2%)			Vaccination status (*n* = 674)	Dose 0	194 (28.9%)	478 (71.1%)	0.414	0.020
	Other	1 (5.6%)	17 (94.4%)				Dose 1	3 (25.0%)	9 (75.0%)		
	Student	7 (20.6%)	27 (79.4%)			Latrine availability (*n* = 800)	No	212 (28.5%)	533 (71.5%)	1.31	0.022
	TDF	3 (30.0%)	7 (70.0%)				Yes	11 (20.0%)	44 (80.0%)		
	Unemployed	13 (31.7%)	28 (68.3%)			Whether HH has undergone disinfection (*n* = 802)	No	214 (40.9%)	309 (59.1%)	23.24	<0.001
							Yes	11 (3.9%)	268 (96.1%)		

Geographical factors played a crucial role, as evidenced by the highly significant association between reported zone and cholera status (*χ*^2^ = 34.16, *p* < 0.001). The Central zone had an alarmingly high infection rate (98.7%) compared to the Northwestern zone (69.2%). Occupation was another significant factor (*χ*^2^ = 30.42, *p* < 0.001), with certain groups, such as children and housewives, showing 100% infection rates. Water source emerged as a critical factor significantly associated with cholera status (*χ*^2^ = 25.74, *p* < 0.001). Spring water users had the highest infection rate (94.1%), followed by hand pump (81.6%) and surface water users (81.5%). Contact with suspected AWD patients (*χ*^2^ = 0.91, *p* = 0.031) and travel history to active areas (*χ*^2^ = 2.84, *p* = 0.042) were also significantly associated with cholera status, although the differences in infection rates were less pronounced.

Regarding diagnostic testing, 602 (75.1%) of the participants underwent rapid diagnostic testing (RDT), while 200 (24.9%) did not. Among those tested, 424 were classified as cholera-positive and 178 as cholera-negative. Among participants who did not undergo RDT testing, 130 were classified as cholera-positive and 70 as cholera-negative. This reflects the use of combined clinical, epidemiological, and laboratory criteria in defining cholera status, rather than reliance on laboratory confirmation alone (Table 2).

**Table 2.** Distribution of rapid diagnostic test (RDT) status by cholera outcome classification (*n* = 802)

RDT status	Cholera status		Total
	Yes	No	
No	130	70	200
Yes	424	178	602
Total	554	248	802

Interestingly, some factors showed counterintuitive associations. Vaccination status was significantly associated with cholera status (*χ*^2^ = 0.414, *p* = 0.020), but those with one dose had a slightly higher infection rate (75.0%) than those with no vaccination (71.1%). Latrine availability was also significantly associated with cholera status (*χ*^2^ = 1.31, *p* = 0.022), with higher crude proportions among households with latrines (80.0%) compared with those without (71.5%). Household disinfection showed a strong association with cholera status (*χ*^2^ = 23.24, *p* < 0.001), with a higher crude proportion of cholera among households that underwent disinfection (96.1%) compared with those that did not (59.1%).

### Parameter estimates from Generalized Estimating Equation (GEE)

#### Risk factors for illness

We fitted a Generalized Estimating Equation model with an exchangeable correlation structure to account for clustering of cases within the 25 reported districts (11 in the Northwestern zone and 14 in the Central zone). The estimated working correlation parameter was high (*α* = 0.949), indicating strong within-district correlation.

The analysis revealed significant associations between demographic, occupational, and environmental factors and cholera cases. Age emerged as a strong predictor, with individuals aged 16–30 years exhibiting twice the odds of cholera compared to the 1- to 15-year reference group (OR = 2.00, *p* < 0.001). Similarly, individuals aged 31–45 years and 46–60 years had elevated odds of cholera (OR = 1.42 and OR = 1.57, respectively, both *p* < 0.001), while the 61+-year group showed no significant association (OR = 1.22, *p* = 0.395). Males had 35% higher odds of cholera compared with females (OR = 1.35, *p* < 0.001).

Occupational disparities were notable and played a crucial role in cholera risk, with gold miners showing a fivefold increase in odds compared to children (OR = 5.01, *p* < 0.001), followed by IDPs (OR = 2.56, *p* < 0.001), farmers (OR = 1.92, *p* < 0.001), and students (OR = 1.79, *p* < 0.001). Household disinfection significantly reduced cholera risk (OR = 0.47, *p* < 0.001), as did sanitation facility availability (OR = 0.90, *p* < 0.001) and vaccination (OR = 0.52, *p* < 0.001).

Environmental factors were strongly associated with cholera occurrence. Compared with hand pump users, spring water users had more than twice the odds of cholera (OR = 2.31, *p* < 0.001), and surface water users had approximately four times higher odds (OR = 4.13, *p* < 0.001). Pipe water use was associated with lower odds, although this was not statistically significant (OR = 0.39, *p* = 0.143). Travel history was a significant risk factor, with those who travelled having 2.39 times higher odds of infection (*p* < 0.01).

#### Subgroup analysis of hotspot and non-hotspot districts

To assess robustness and determine whether the findings were driven primarily by the most affected districts, we conducted a subgroup GEE analysis comparing hotspot districts (Asgede and Endabaguna Town) with the remaining affected woredas (Table 3). Overall, the direction of associations remained consistent across both subgroups, although the magnitude of effects was generally stronger in hotspot districts. Risk factors related to water source, occupation, and travel history showed more pronounced associations in hotspot areas, indicating intensified transmission dynamics in these districts. Similarly, protective factors such as household disinfection, latrine availability, and vaccination demonstrated consistent effects across both subgroups, with slightly stronger protective patterns observed in hotspot districts. These findings suggest that the identified determinants of cholera transmission are robust across the study area, while the intensity of their effects varies between hotspot and non-hotspot settings.

**Table 3.** Subgroup GEE analysis comparing hotspot and non-hotspot districts

Predictor	Hotspot districts (Asgede ± Endabaguna) aOR (95% CI)	*p*-value	Non-hotspot districts aOR (95% CI)	*p*-value
Male sex	1.42 (1.05–1.92)	0.021	1.31 (1.07–1.60)	0.009
Age 16–30 vs. 1–15	2.28 (1.54–3.38)	<0.001	1.88 (1.41–2.50)	<0.001
Gold miner occupation	6.12 (3.40–11.02)	<0.001	3.41 (2.05–5.66)	<0.001
Internally displaced person (IDP)	3.05 (1.74–5.33)	<0.001	2.12 (1.33–3.37)	0.002
Surface water source	5.02 (3.01–8.39)	<0.001	3.62 (2.45–5.36)	<0.001
Spring water source	2.84 (1.66–4.86)	<0.001	2.08 (1.41–3.06)	<0.001
Travel history to active area	2.91 (1.93–4.40)	<0.001	2.08 (1.53–2.83)	<0.001
Household disinfection	0.39 (0.26–0.58)	<0.001	0.54 (0.41–0.71)	<0.001
Latrine availability	0.86 (0.78–0.95)	0.004	0.91 (0.84–0.99)	0.028
Cholera vaccination (≥1 dose)	0.49 (0.34–0.70)	<0.001	0.55 (0.41–0.74)	<0.001

#### Model comparison and fitting

We compared results from the Generalized Estimating Equations model with those from standard logistic regression. The GEE model accounted for clustering of cases within districts and showed strong within-district correlation (working correlation *α* = 0.95). Robust Wald tests from the GEE model (Table 4) identified age 16–30 years (OR = 2.05, 95% CI: 1.55–2.71), age 31–45 years (OR = 1.46, 95% CI: 1.10–1.94), male sex (OR = 1.34, 95% CI: 1.10–1.63), surface water use (OR = 4.10, 95% CI: 2.80–6.05), spring water use (OR = 2.30, 95% CI: 1.55–3.40), travel history (OR = 2.35, 95% CI: 1.70–3.26), and occupations such as gold miner (OR = 5.00, 95% CI: 3.00–8.40) and IDP (OR = 2.60, 95% CI: 1.60–4.10) as factors associated with higher odds of cholera. Vaccination (OR = 0.52, 95% CI: 0.38–0.71) and latrine availability (OR = 0.90, 95% CI: 0.84–0.97) were associated with lower odds.

**Table 4.** Parameter estimates, adjusted odds ratios, and 95% CI from GEE model (exchangeable correlation)

Predictor	Estimate (*β*)	Std. Err	Adjusted OR (eβ)	95% CI for OR	Wald	*p*-value	
Age group (Ref: 1–15 yrs)						
16–30 yrs	0.69	0.28	1.99	1.15–3.43	0.29	<0.001
31–45 yrs	0.35	0.16	1.42	1.03–1.96	0.39	<0.001
46–60 yrs	0.45	0.19	1.57	1.08–2.28	0.83	<0.001
61+ yrs	0.20	0.24	1.22	0.77–1.94	0.01	0.395
Sex (Ref: Female)						
Male	0.30	0.10	1.35	1.10–1.65	0.16	<0.001
Travel history (Ref: No)						
Yes	0.87	0.17	2.39	1.70–3.37	0.99	<0.001
Vaccination (Ref: Unvaccinated)						
1 Dose	−0.65	0.19	0.52	0.36–0.75	1.76	<0.001
Latrine availability (Ref: No)						
Yes	−0.11	0.04	0.9	0.84–0.97	0.35	<0.001
Household disinfection (Ref: No)						
Yes	−0.75	0.18	0.47	0.33–0.67	2.33	<0.001
Contact with AWD case (Ref: No)						
Yes	−0.65	0.21	0.52	0.35–0.78	1.07	<0.001
Occupation (Ref: Child)						
Farmer	0.65	0.2	1.92	1.30–2.80	0.14	<0.001
Gold miner	1.61	0.31	5.01	2.70–9.30	13.06	<0.001
Herdman	0.17	0.15	1.19	0.89–1.59	11.13	<0.001
Housewife	0.15	0.18	1.16	0.82–1.65	6.28	<0.001
IDP	0.94	0.24	2.56	1.60–4.10	0.17	<0.001
Student	0.58	0.19	1.79	1.10–2.90	0.18	<0.001
TDF	0.41	0.31	1.51	0.82–2.80	0.02	0.186
Unemployed	0.26	0.12	1.3	1.03–1.63	0.22	<0.001
Other	0.63	0.22	1.88	1.22–2.90	3.95	<0.001
Water source (Ref: Hand pump)						
Pipe water	−0.94	0.64	0.39	0.11–1.35	0.02	0.143
River water	0.4	0.12	1.49	1.18–1.88	0.21	<0.001
Spring water	0.84	0.20	2.31	1.55–3.40	16.41	<0.001
Surface water	1.42	0.20	4.13	2.80–6.05	0.32	<0.001
Other	−0.24	0.18	0.79	0.56–1.12	0.02	0.185

The standard logistic regression analysis produced results that were consistent in direction and magnitude with the GEE model (Supplementary Table S1). However, standard errors were generally smaller, and some additional predictors appeared statistically significant, reflecting the lack of adjustment for within-district clustering. Model performance was comparable between approaches, with similar discrimination and calibration (GEE: AUC = 0.83, Brier score = 0.16; logistic regression: AUC = 0.78, Brier score = 0.18). Multicollinearity was low across predictors (all VIF < 3). Sensitivity analyses showed consistent results across model specifications, with no meaningful changes in the direction or statistical significance of the main predictors.

## Discussion

This study aimed to characterize the epidemiology, spatial distribution, and determinants of the 2024 cholera outbreak in the most affected accessible zones of Tigray, Ethiopia, using surveillance data and multivariable modelling. Overall, the findings showed strong geographic clustering of cholera cases, substantial heterogeneity in attack rates across districts, and important associations with age, sex, occupation, water source, travel history, sanitation, household disinfection, and vaccination. The results highlight the combined influence of environmental exposure, mobility, and WaSH conditions in shaping outbreak dynamics.

Some findings from the crude (univariate) analysis appeared counterintuitive, particularly the higher proportions of cholera among individuals living in households with latrines, those reporting household disinfection, and vaccinated individuals. These patterns are best explained by confounding and outbreak response dynamics. During the epidemic, interventions such as vaccination and household disinfection were implemented reactively and prioritized in high-transmission districts and among households already affected by cholera. As a result, these interventions were more common among populations with elevated baseline exposure, leading to misleading crude associations. In addition, reverse causation likely contributed to the observed pattern for household disinfection, which was typically conducted after case identification. After adjusting for clustering and confounding in the multivariable analysis, these factors showed protective effects consistent with established evidence. This highlights the importance of multivariable modelling when interpreting observational data collected during outbreak response settings.

The spatial heterogeneity in attack rates, as demonstrated by the significant disparities between districts, highlights the importance of localized responses. For instance, the alarmingly high attack rates in Northwestern Tigray, notably in *Asgede* and *Endabaguna Town*, indicate a critical need for intensified public health measures in these regions. Such measures may include improved access to clean water, enhanced sanitation infrastructure, and targeted vaccination campaigns. Similar approaches have proven effective in other contexts, such as the cholera control programmes implemented in Haiti [32].

The demographic and environmental determinants identified in this study align with previous research on cholera risk factors. For example, the elevated risk among individuals aged 16–30 years and occupational groups such as gold miners and farmers suggests that mobility and exposure to contaminated environments are key drivers of transmission. Studies in similar settings, such as Nigeria and Bangladesh, have highlighted comparable patterns of vulnerability linked to occupation and age [33].

Household disinfection, when effectively implemented, was shown to significantly reduce cholera risk. This finding aligns with studies from regions such as South Asia and Sub-Saharan Africa, where targeted disinfection campaigns during outbreaks reduced disease transmission [34, 35]. The observed efficacy underscores the importance of equipping households with appropriate disinfectants and educating communities on proper usage. Additionally, community-led disinfection programmes could amplify these benefits, particularly in areas with limited access to healthcare facilities.

Sanitation facility availability was another critical factor associated with reduced cholera risk. This reinforces the role of sustained investments in sanitation infrastructure as a cornerstone of cholera prevention. Studies from Kenya and India have demonstrated that increasing access to latrines and promoting their use can significantly lower disease burden in vulnerable populations [36]. Scaling up such interventions in high-risk districts could yield substantial public health benefits.

Vaccination emerged as an effective intervention, with vaccinated individuals exhibiting a lower risk of cholera infection. This finding is consistent with global evidence supporting the efficacy of oral cholera vaccines (OCVs) in outbreak settings [37, 38]. The results emphasize the importance of incorporating vaccination into integrated cholera control strategies, particularly in endemic regions like Tigray. Expanding vaccine coverage and ensuring timely administration during outbreaks could serve as a powerful tool in mitigating cholera’s impact.

Water source emerged as one of the strongest determinants of cholera occurrence. Reliance on spring and surface water was associated with substantially higher odds of cholera compared with hand pump use, mirroring findings from Zimbabwe and Yemen. In our study, pipe water showed a protective direction of effect but was not statistically significant [35, 39]. However, substantial evidence demonstrates that access to safely managed and piped water reduces cholera transmission. For example, a systematic review by Fewtrell et al. showed that improved water supply and quality interventions significantly reduce diarrhoeal diseases [40]. Similarly, Ali et al. highlighted unsafe water as a key driver of cholera burden globally, with improved water infrastructure associated with reduced risk [39]. In addition, the World Health Organization emphasizes access to safe drinking water as a cornerstone of cholera prevention and control [41]. These findings reinforce the central role of water, sanitation, and hygiene interventions in outbreak prevention and control.

### Limitations

This study has several limitations that should be considered when interpreting the findings. First, the number of cholera cases captured in the surveillance line list likely underestimates the true burden of disease. Surveillance relied primarily on health facility reporting and active case finding in accessible areas. Individuals living in remote locations, IDPs, those with limited healthcare access, and individuals who died before reaching a health facility may have been missed. This potential under-ascertainment may have introduced selection bias and could affect the estimated magnitude of associations. Second, the study included only two of the six cholera-affected accessible zones of Tigray (Central and Northwestern). Western Tigray remained inaccessible due to security constraints, and the remaining accessible zones were not included in this analysis because the outbreak was most intense in the selected zones. Surveillance activities, laboratory confirmation, and active case finding could not be conducted in inaccessible areas, and cholera transmission dynamics in other zones may differ. As a result, the spatial distribution and overall burden reported here may underestimate the true magnitude of the outbreak across the entire region, and the findings may not be fully generalizable to all zones of Tigray. Differences in surveillance intensity, access to health services, and response prioritization between zones may also have influenced the observed differences in cholera rates. During the outbreak, areas with higher transmission or better access may have received more intensive case finding and reporting. Consequently, part of the variation observed between zones may reflect differences in surveillance and reporting rather than true differences in transmission alone. Third, residual confounding may remain despite the use of multivariable Generalized Estimating Equations. Although we adjusted for key demographic, environmental, and behavioural variables, unmeasured factors such as nutritional status, crowding, population mobility, and detailed water quality indicators may have influenced cholera occurrence. The use of multivariable modelling and clustering adjustments helped minimize confounding, but it cannot be completely eliminated. Fourth, the observational nature of the data limits causal inference. The study identifies associations rather than causal relationships. To mitigate this limitation, we used multivariable modelling, cluster-adjusted standard errors, and sensitivity analyses to strengthen the robustness of the findings. Fifth, measurement error may be present because some variables were collected during an emergency response setting. Information such as water source use, travel history, and household disinfection relied partly on self-report and routine surveillance records, which may be subject to recall or reporting bias. Such misclassification could attenuate or overestimate associations. Finally, the study included 25 districts as clusters in the Generalized Estimating Equations analysis, whereas GEE typically performs best with larger numbers of clusters. With fewer clusters, standard errors may be underestimated, which could increase the risk of type I error. To mitigate this concern, we used robust (sandwich) standard errors and conducted sensitivity analyses, including comparison with standard logistic regression models. The consistency of direction and magnitude of the main associations across modelling approaches provides reassurance regarding the robustness of the findings. Despite these limitations, the study provides important evidence on cholera epidemiology in a conflict-affected setting and offers valuable insights for targeted public health interventions.

## Supporting information

10.1017/S0950268826101514.sm001 Gebremeskel et al. supplementary material Gebremeskel et al. supplementary material

## Data Availability

The data that support the findings of this study are not openly available due to reasons of sensitivity and are available from the corresponding author upon reasonable request. Data are located in controlled access data storage at Tigray Health Research Institute (https://thri.gov.et/about/).

## Abbreviation

AWD: acute watery diarrhoea
CFR: case fatality rate
CLL: cholera line list
CTC: Cholera Treatment Centre
IDP: internally displaced person
OCV: oral cholera vaccine
OR: odds ratio
PHEM: Public Health Emergency Management
PHEOC: Public Health Emergency Operation Centre
TRHB: Tigray Regional Health Bureau
WaSH: water sanitation and hygiene
WHO: World Health Organization

## Supplementary material

The supplementary material for this article can be found at http://doi.org/10.1017/S0950268826101514.

## Patient and public involvement

Patients and/or the public were not involved in the design, or conduct, or reporting, or dissemination plans of this research.
