# Supplementary material for: Epidemiological evaluation of cholera outbreak dynamics in conflict-affected Central and Northwestern Tigray, northern Ethiopia: evidence from GEE analysis
Source: Epidemiol Infect. 2026 Apr 30;154:e64. doi: 10.1017/S0950268826101514 (PMC13200026; doi:10.1017/S0950268826101514)
Supplement: Gebremeskel et al. supplementary material [file S0950268826101514sup001.docx]

**Supplementary Table S1. Results of standard logistic regression analysis**

| **Predictor** | **Adjusted OR** | **95% CI** | **p-value** |
| --- | --- | --- | --- |
| **Age group (Ref: 1–15 yrs)** | | |  |
| *16–30 yrs* | *2.15* | *1.30 – 3.55* | *<0.001* |
| *31–45 yrs* | *1.50* | *1.10 – 2.05* | *0.008* |
| *46–60 yrs* | *1.62* | *1.12 – 2.35* | *0.005* |
| *61+ yrs* | *1.28* | *0.82 – 2.00* | *0.270* |
| **Sex (Ref: Female)** | |  |  |
| *Male* | *1.4* | *1.15 – 1.70* | *<0.001* |
| **Travel history (Ref: No)** | | |  |
| *Yes* | *2.55* | *1.85 – 3.50* | *<0.001* |
| **Vaccination (Ref: Unvaccinated)** | | | |
| *≥1 dose* | *0.55* | *0.40 – 0.75* | *<0.001* |
| **Latrine availability (Ref: No)** | | |  |
| *Yes* | *0.88* | *0.82 – 0.95* | *0.002* |
| **Household disinfection (Ref: No)** | | | |
| *Yes* | *0.45* | *0.32 – 0.63* | *<0.001* |
| **Contact with AWD case (Ref: No)** | | | |
| *Yes* | *0.58* | *0.40 – 0.85* | *0.004* |
| **Occupation (Ref: Child)** | | |  |
| *Farmer* | *2.00* | *1.40 – 2.90* | *<0.001* |
| *Gold miner* | *5.40* | *3.10 – 9.50* | *<0.001* |
| *Herdman* | *1.25* | *0.95 – 1.65* | *0.110* |
| *Housewife* | *1.20* | *0.90 – 1.70* | *0.210* |
| *IDP* | *2.70* | *1.70 – 4.20* | *<0.001* |
| *Student* | *1.85* | *1.15 – 3.00* | *0.012* |
| *Unemployed* | *1.35* | *1.05 – 1.70* | *0.020* |
| *Other* | *1.95* | *1.30 – 3.00* | *0.002* |
| **Water source (Ref: Hand pump)** | | | |
| *Pipe water* | *0.42* | *0.12 – 1.45* | *0.170* |
| *River water* | *1.55* | *1.25 – 1.95* | *<0.001* |
| *Spring water* | *2.45* | *1.70 – 3.55* | *<0.001* |
| *Surface water* | *4.40* | *3.05 – 6.35* | *<0.001* |
| *Other* | *0.82* | *0.60 – 1.15* | *0.240* |
